# Synergistic activity of fosfomycin–meropenem and fosfomycin–colistin against carbapenem resistant *Klebsiella pneumoniae*: an *in vitro* evidence

**DOI:** 10.2144/fsoa-2019-0074

**Published:** 2020-02-26

**Authors:** Yamuna Devi Bakthavatchalam, Abirami Shankar, Dhiviya Prabaa Muthuirulandi Sethuvel, Kalaiarasi Asokan, Kalaiarasi Kanthan, Balaji Veeraraghavan

**Affiliations:** 1 Department of Clinical Microbiology, Christian Medical College, Vellore 632004, India

**Keywords:** colistin, fosfomcyin, fosfomycin–colistin, fosfomycin–meropenem, *Klebsiella pneumoniae*, meropenem, NDM, OXA-48-like, time-kill assay

## Abstract

**Aim:**

To evaluate the antibacterial activity of fosfomycin–meropenem and fosfomycin–colistin combinations against carbapenem-resistant *Klebsiella pneumoniae* (CR-Kp).

**Methods:**

A total of 50 CR-Kp isolates recovered from blood cultures were included in this study. All the CR-Kp isolates were screened for the presence of carbapenem resistant genes *bla*_IMP_. *bla*_VIM_. *bla*_NDM_. *bla*_OXA-48_ like, *bla*_KPC_. *bla*_GES_.#x00A0;and *bla*_SPM_. Combination testing of fosfomycin–meropenem and fosfomycin–colistin were performed using time-kill assay.

**Results:**

Fosfomycin–meropenem combination showed synergy in 20% of the tested CR-Kp isolates. While, fosfomycin–colistin exhibited synergy against 16% of the isolates. A total of 68% (n = 34) of CR-Kp isolates were characterised as OXA-48-like producers and 22% (n = 11) as NDM producers. Synergistic activity of these combinations was observed against OXA-48, NDM and NDM + OXA-48 co-producers.

**Conclusion:**

Considerable synergistic antibacterial activity of fosfomycin–meropenem and fosfomycin–colistin was not observed against CR-Kp isolates. Therefore, these combinations may not be promising for infections associated with CR-Kp.

## Lay abstract

Carbapenem-resistant *Klebsiella pneumoniae* (CR-Kp) infections are difficult to treat and are associated with a high mortality rate. This study aimed to evaluate the synergistic activity of fosfomycin–meropenem and fosfomycin–colistin combinations against CR-Kp. Synergistic activity of these combinations was observed against OXA-48, NDM and NDM + OXA-48 co-producers. However, synergism was not found to be significant. Therefore, these combinations may not be promising for infections associated with CR-Kp.

Carbapenem-resistant *Enterobacteriaceae* (CRE) infections represent an increasing global threat. CRE infections are highly endemic in India, but an estimate of the infection burden is lacking. Currently, double or triple combination therapy based on carbapenem, polymyxins and tigecycline is very often prescribed for the treatment of CRE infections. Monotherapy with either colistin, fosfomycin or tigecycline is insufficient, which contributes to a modest response in patients. Emergence and an increasing trend of colistin resistance among CRE are alarming. The Clinical and Laboratory Standards Institute (CLSI) guidelines do not recommend fosfomycin minimum inhibitory concentration (MIC) breakpoints for *Enterobacteriaceae*, except *Escherichia coli* [1]. However, the European Committee on Antimicrobial Susceptibility Testing guidelines have recommended fosfomycin MIC breakpoints for *Enterobacteriaceae* [2].

Fosfomycin is an alternative for the treatment of CRE infections, especially when combined with other antibiotics, due to its synergistic effect. Fosfomycin prevents the transpeptidation of peptidoglycan in bacteria [3]. Similar to other beta-lactams, meropenem inhibits peptidoglycan synthesis by binding to penicillin-binding protein 2, 3 and 4 [4]. Meanwhile, colistin binds to lipopolysaccharide and phospholipids in the outer cell membrane of Gram-negative bacteria [5]. This differentiated mechanism of action has been proposed to enhance killing when fosfomycin is combined with meropenem and colistin. Studies have documented the synergistic activity of fosfomycin with various antibacterial agents. However, limited information is available on the antibacterial activity of fosfomycin–meropenem and fosfomycin–colistin against extreme drug-resistant *K. pneumoniae*. The present study aimed to investigate the antibacterial activity of fosfomycin combined with meropenem and colistin against carbapenem-resistant *K. pneumoniae* (CR-Kp) using time-kill assay.

## Material & methods

### Bacterial strains

Nonduplicate isolates of CR-Kp (n = 50) recovered from blood cultures obtained during 2017–2018 were included. These blood cultures were received for routine laboratory diagnosis. All the data or samples were fully anonymized before accessing them for further processing. This study only utilized isolates from positive blood cultures. Moreover, the study does not involve patients’ direct participation or follow-up. The study was conducted at the 2600-bed tertiary care hospital, Christian Medical College in Vellore, India.

### Antimicrobial susceptibility testing

The MIC of meropenem and colistin was determined using the broth microdilution method (CLSI, 2015) [6]. The agar dilution method was used for the determination of fosfomycin MIC (CLSI, 2015) [6]. Cation-adjusted Mueller-Hinton broth-containing 25 μg/ml of glucose-6-phosphate was used for fosfomycin susceptibility testing. A meropenem MIC of ≥4 μg/ml was considered as resistant [6]. The European Committee on Antimicrobial Susceptibility Testing-recommended breakpoints were used for the interpretation of fosfomycin and colistin susceptibility [2]. An isolate which had fosfomycin MIC of >32 μg/ml and colistin MIC of >2 μg/ml was considered as resistant. For fosfomycin susceptibility testing, *E.coli* ATCC 25922 was used as the quality control strain. For colistin susceptibility testing, *E. coli* ATCC 25922, *P. aeruginosa* ATCC 27853 and colistin-resistant *E. coli* (*mcr*-1-positive) were used as the control strains.

### Molecular detection of resistance genes

Multiplex PCR was performed for the detection of carbapenem-resistant genes *bla*_IMP_. *bla*_VIM_. *bla*_NDM_. *bla*_OXA-48_ like, *bla*_KPC_. *bla*_GES_.#x00A0;and *bla*_SPM_ [7–9].

### Time kill assays

Time kill assay (TKA) was performed as previously described [10]. The combinations of fosfomycin plus meropenem and fosfomycin plus colistin were tested against CR-Kp isolates. The Mueller-Hinton broth-containing 25 μg/ml of glucose-6-phosphate was used for combination testing. For TKA assays, the initial inoculum was targeted at 1 × 10^6^ colony forming unit (CFU)/ml. A sampling of the inoculum incubated with fosfomycin, meropenem, colistin, fosfomycin plus meropenem and fosfomycin plus colistin were performed at times of 0, 3, 6 and 24 h of postincubation at 35 ± 2°C. The inoculum was diluted 1 in 100 using a 0.85% saline solution. A volume of 100 μl was plated directly onto nutrient agar plates and incubated at 37°C for 24 h. After incubation, colonies were counted and expressed as log_10_ CFU/ml. The viable count was plotted over time to create time-kill curves. For each batch of testing, sterility and growth control (without antibiotic) were included. TKA interpretation was performed as described earlier by Isenberg [10]. Synergy was defined as a ≥2 log_10_ CFU/ml decrease in combination compared with the most active single agent; indifference was defined as a <2-log_10_ CFU/ml increase or decrease in colony count at 6 or 24 h in combination compared with the most active single agent; antagonism was defined as a ≥2 log_10_ CFU/ml increase in combination compared with the most active single agent; bactericidal activity was defined as a ≥3 log_10_ CFU/ml reduction from the initial inoculum; regrowth was defined as an initial decrease of ≥3 log_10_ CFU/ml followed by ≥2 log_10_ CFU/ml increase at 24 h. For each batch of testing, *Escherichia coli* ATCC 25922 was used as the quality control strains.

## Results

All the tested *K. pneumoniae* isolates were resistant to meropenem with the MICs ranging from 4 to 512 μg/ml. Colistin MICs ranges from 0.5 to 128 μg/ml, while fosfomycin MICs ranges from 2 to 256 μg/ml. Among the tested CR-Kp isolates, 48% (n = 24) were resistant to fosfomycin and 30% (n = 14) were resistant to colistin. Eight CR-Kp isolates were resistant to both colistin (16–128 μg/ml) and fosfomycin (64–256 μg/ml). For each batch of testing, *mcr*-1-positive *E. coli* strain produced a consistent colistin MIC of 4 μg/ml. All CR-Kp isolates were screened for the presence of carbapenem-resistant genes. Majority, 68% (n = 34) of CR-Kp isolates carried OXA-48-like gene and 22% (n = 11) was found with NDM gene. Co-existence of OXA-48-like and NDM genes were seen in five *K.pneumoniae* isolates.

**Figure 1.**
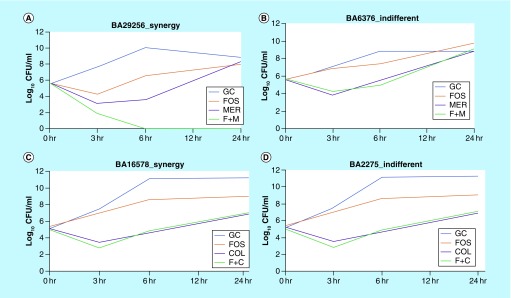
Time-kill curves of fosfomycin–meropenem and fosfomycin–colistin combination. **(A)** CR-Kp isolate showing synergy with fosfomcyin–meropenem combination; **(B)** isolate exhibits indifferent activity against fosfomcyin–meropenem; **(C)** and **(D)** isolate showing synergy and indifferent activity against fosfomycin–colistin combination. C: Colistin; col: Colistin; CR-Kp: Carbapenem-resistant *Klebsiella pneumoniae*; Fos: Fosfomycin; F: Fosfomycin; GC: Growth control; Mer: Meropenem.

TKA experiments were performed for all CR-Kp (n = 50) isolates. Fosfomycin–meropenem combination showed synergistic antibacterial activity in 20% (n = 10) of the isolates (Table 1). Meanwhile, fosfomycin–colistin showed synergistic activity in 16% (n = 8) of CR-Kp isolates. A representative time-kill curves of these two combinations are shown in Figure 1. At 24 h of postincubation, regrowth of the strains was observed with both the combinations (Table 1). Retesting of MIC in these isolates showed a two to sixfold increase in fosfomycin MICs, while colistin MICs remains unchanged.

**Table 1.** Synergy, indifference, bactericidal effect and regrowth of fosfomcyin–meropenem and fosfomycin–colistin combination against carbapenem resistant *K. pneumoniae* in time kill assay.

Tested antibiotic combinations	n (%)					
	Synergy	Indifference	Bactericidal effect		Regrowth	
			Positive	Negative	Positive	Negative
Fosfomycin–meropenem	10 (20)	40 (80)	Syn – 5 (50); Ind – 16 (40)	Syn – 5 (50); Ind – 24 (60)	Syn – 3 (30); Ind – 22 (55)	Syn – 7 (70); Ind – 18 (45)
Fosfomycin–colistin	8 (16)	42 (84)	Syn – 4 (50); Ind – 15 (36)	Syn – 4 (50); Ind – 27 (64)	Syn – 0 (0); Ind – 25 (60)	Syn – 8 (100); Ind – 17 (40)

**Figure 2.**
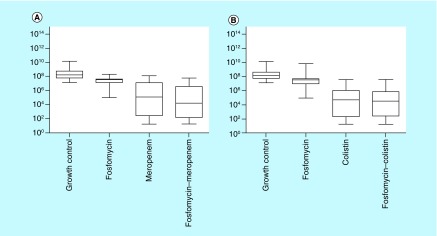
Box plot showing median log_10_ CFU/ml of carbapenem resistant *K. pneumoniae*. **(A)** Box plot showing median log_10_ CFU/ml of CR-Kp observed with fosfomycin, meropenem and fosfomycin–meropenem combination. **(B)** Box plot showing median log_10_ CFU/ml of CR-Kp observed with fosfomycin, colistin and fosfomycin–colistin combination. CFU: Colony-forming unit; CR-Kp: Carbapenem-resistant *Klebsiella pneumoniae*.

Considerable reduction in log_10_ CFU/ml was observed with the fosfomycin–meropenem combination than fosfomycin or meropenem alone (Figure 2). However, such a difference in the median log_10_ CFU/ml was not seen between fosfomycin–colistin combination and colistin alone. Antagonism was not observed in either of these tested combinations.

Synergistic activity of fosfomycin–meropenem and fosfomycin–colistin was observed against NDM, OXA-48-like and NDM plus OXA-48-like co-producers (Table 2). However, this effect was not significantly seen.

**Table 2.** Combination testing of fosfomcyin–meropenem and fosfomcyin–colistin against carbapenemase producing *K. pneumoniae* using time-kill assay.

Carbapenem resistant genes (n)	Fosfomcyin–meropenem		Fosfomcyin–colistin	
	Synergy, n (%)	Indifference, n (%)	Synergy, n (%)	Indifference, n (%)
*bla*_OXA-48_.like (n = 34)	8 (24)	26 (76)	5 (15)	29 (85)
*bla*_NDM_ (n = 11)	1 (9)	10 (91)	2 (18)	9 (82)
*bla*_OXA-48_.like + *bla*_NDM_ (n = 5)	1 (20)	4 (80)	1 (20)	4 (80)

## Discussion

Infections associated with CRE are of great concern and associated with poor outcome. However, optimal management of CRE infections remains unclear. In recent years, fosfomycin and colistin have gained attention in treating multidrug-resistant and extreme drug-resistant Gram-negative infections. Suboptimal dose and prolonged monotherapy with colistin contribute to the development of colistin resistance [11]. Fosfomycin has a unique chemical structure and mechanism of action and thus cross-resistance is uncommon. The present study aimed to evaluate the antibacterial activity of fosfomycin–meropenem and fosfomycin–colistin combinations against CR-Kp isolates.

In intensive care units, an optimal colistin dosing regimen against CRE infections remains uncertain. Higher doses of colistin are associated with the potential risk of developing nephrotoxicity in patients [12]. Colistin penetrates poorly into the lung tissues and achieves lower concentration in the pleural cavity [13,14]. In contrast, intravenous infusion of fosfomycin has been reported with a high plasma concentration and greater tissue penetration [15]. In the infected lung tissue mouse model, intravenous administration of fosfomycin showed good penetration and achieves higher concentration in the pleural fluid [16]. Fosfomycin is not associated with the risk of nephrotoxicity [17].

In recent years, colistin and fosfomycin are being frequently used to treat carbapenem-resistant Gram-negative infections. An *in vitro* study has demonstrated that 78% of CRE isolates were susceptible to fosfomycin [18]. In the present study, 58% of CR-Kp isolates were susceptible to fosfomycin. In addition, co-existence of resistance to colistin and fosfomycin was noticed in 16% of the tested isolates. Further, these isolates were not investigated for molecular determinants. Presence of *fos*A gene and mutation in *mgr*B has been described for the co-occurence of fosfomycin and colistin resistance in CR-Kp [19].

Studies have reported an *in vitro* synergy of fosfomycin with β-lactams, carbapenems and colistin against CRE infections [20–22]. A higher percentage of fosfomycin synergy has been described with carbapenems (70%) than colistin (36%) against CR-Kp isolates [23]. It is perhaps notable that synergy with fosfomycin–meropenem and fosfomycin–colistin combinations was not observed in our study. This could be due to the predominance of OXA-48-like producers, showing resistance to fosfomycin and colistin in *K. pneumoniae*. A study has demonstrated the synergistic activity of fosfomycin–meropenem combination against fosfomycin-resistant CR-Kp [24]. Similarly, in the present study, synergistic activity of fosfomycin–meropenem combination was noted against colistin- and fosfomycin-resistant CR-Kp.

Synergistic antibacterial activity of colistin and fosfomycin has been reported against *K. pneumoniae* carbapenemase (KPC)-producing *K. pneumoniae* and NDM-1 producing *Enterobacteriaceae* [25,26] while fosfomycin plus colistin combination has been reported with antagonistic activity against OXA-48 producing *K. pneumoniae* [27]. In contrast, in our study, synergistic activity of fosfomcyin-colistin combination was observed against OXA-48-like producers.

The present study showed synergistic activity of fosfomycin–colistin and fosfomycin–meropenem combinations against OXA-48-like, NDM and co-producers. However, remarkable synergy is not observed against these carbapenemase-producing *K. pneumoniae*. This could be due to the fact that the *fos*A gene is chromosomally encoded in *K. pneumoniae* [28] and mutations in GlpT (glycerol-3-phosphate [G3P] transporter) and UhpT (glucose-6-phosphate [G6P] transporter) are the most frequent events leading to lowered fosfomycin susceptibility in *K. pneumoniae* [29].

In pharmacodynamic studies, resistant subpopulations suppression with fosfomycin–meropenem and fosfomycin–colistin combinations has been reported [25,30]. A clinical cure rate of 70% was reported in patients treated with fosfomycin–meropenem combination, who had colistin-resistant Gram-negative infections [31]. Similarly, in the present study, fosfomycin–meropenem had better synergistic activity against colistin-resistant CR-Kp than fosfomycin–colistin combination.

## Conclusion & future perspective

In conclusion, the present study revealed a higher resistance rate to fosfomycin and colistin in *K. pneumoniae*. Synergistic activity of both fosfomycin–meropenem and fosfomycin–colistin combinations was demonstrated against NDM, OXA-48-like producers and co-producers of NDM plus OXA-48-like. However, the synergistic activity was not found to be remarkable. Therefore, these combinations may not be promising for treating CR-Kp infections.

CR-Kp infections are difficult to manage because of the limited treatment options. Combination therapy is often preferred to treat CR-Kp. NDM and OXA-48-producing CR-Kp are expanding globally. A combination of fosfomycin with meropenem or colistin is not considerably promising in treating CR-Kp infections. A novel approach of ceftazidime-avibactam in combination with aztreonam would be a promising option. In the future, novel antibiotics including cefipime tazobactam, cefiderocol and ervacycline will be more beneficial in treating carbapenem-resistant infections.

**Executive summary**

- Carbapenem resistant carbapenem-resistant *Klebsiella pneumoniae* infections are difficult to treat and are associated with high mortality and morbidity.
- Synergistic activity of fosfomycin–meropenem and fosfomycin–colistin combination was observed in 20 and 16% of carbapenem-resistant *Klebsiella pneumoniae* isolates, respectively.
- Antagonism was not observed with these combinations.
- Synergistic activity of these combinations were seen against NDM, OXA-48 and co-producers.
